# Examination of the Cumulative Risk Assessment and Nutritional Profiles among College Ballet Dancers

**DOI:** 10.3390/ijerph20054269

**Published:** 2023-02-28

**Authors:** Kenya Moore, Nancy A. Uriegas, Jessica Pia, Dawn M. Emerson, Kelly Pritchett, Toni M. Torres-McGehee

**Affiliations:** 1 Department of Exercise Science, Arnold School of Public Health, University of South Carolina, Columbia, SC 29208, USA; 2 Department of Health Sciences, Central Washington University, Ellensburg, WA 98926, USA

**Keywords:** female athlete triad, dance, nutrition

## Abstract

This study examined female collegiate ballet dancers’ (*n* = 28) Female Athlete Triad (Triad) risk via the Cumulative Risk Assessment (CRA) and nutritional profiles (macro- and micronutrients; *n* = 26). The CRA identified Triad return to play criteria (RTP: Full Clearance, Provisional Clearance, or Restricted/Medical Disqualified) by assessing eating disorder risk, low energy availability, menstrual cycle dysfunction, and low bone mineral density. Seven-day dietary assessments identified any energy imbalances of macro- and micronutrients. Ballet dancers were identified as low, within normal, or high for each of the 19 nutrients assessed. Basic descriptive statistics assessed CRA risk classification and dietary macro- and micronutrient levels. Dancers averaged 3.5 ± 1.6 total score on the CRA. Based on these scores, the RTP outcomes revealed Full Clearance 7.1%, *n* = 2; Provisional Clearance 82.1%, *n* = 23; and Restricted/Medical Disqualification 10.7%, *n* = 3. Dietary reports revealed that 96.2% (*n* = 25) of ballet dancers were low in carbohydrates, 92.3% (*n* = 24) low in protein, 19.2% (*n* = 5) low in fat percent, 19.2% (*n* = 5) exceeding saturated fats, 100% (*n* = 26) low in Vitamin D, and 96.2% (*n* = 25) low in calcium. Due to the variability in individual risks and nutrient requirements, a patient-centered approach is a critical part of early prevention, evaluation, intervention, and healthcare for the Triad and nutritional-based clinical evaluations.

## 1. Introduction

Ballet is a physical art form that places major emphasis on aesthetic appearance and movements of the body and is a difficult form of dance in how it challenges the musculoskeletal system [1]. It takes many years to master the skills required to perform ballet, and it is a mixture of low- and high-intensity movements with quick changes in direction over long durations. Dancers must possess the ability to reserve power for explosive jumps but also maintain endurance [2]. Differing from the traditional athlete, dancers must not only be strong but elegant, and evoke emotion while maintaining balance and control in confined areas. Performing artists seeking to achieve optimal performance must not only emphasize training but their overall nutritional status. While general stressors of a dancer can vary from classes, performances, changes in surfaces, and psychological elements, we must also consider how nutritional imbalances in this setting can yield detrimental results [3]. The framework of dietary consumption is vital, and although low energy availability (LEA) can stem from over-exercising, it is often due to intentional or unintentional dietary restrictions [4]. Specifically, in dancers, consistent insufficiencies with energy availability (EA) have been documented [5,6]. The culture of dance may also create atmospheres impeding proper discussion about nutrition and incite a dancer’s fears surrounding food intake, especially around instructors. Nutritional education strategies are important for maximizing performance outcomes and supporting healthier dietary practices throughout life [7].

Generally, all major nutrients can be obtained through a well-balanced diet of macro- and micronutrients. Macronutrients (i.e., carbs, protein, and fat) serve as the primary source of fuel, while micronutrients include both vitamins and minerals (e.g., iron, calcium, etc.) that aid in supporting the detailed metabolic processes [8]. It is not uncommon to find energy availability deficits that are coupled with poor nutritional intake. This may lead to LEA, increasing the risk of the onset of metabolic consequences of the Female Athlete Triad (Triad). A patient-centered approach, however, beyond focusing solely on the dietary needs of performing artists, is to actually explore the individual components of the Triad. The Triad, often viewed along a spectrum, is a combination of LEA with or without an eating disorder (ED), menstrual dysfunction, and impaired bone health [9,10]. Each can occur alone or coexist [11]; for example, in a sample of physically active females, the prevalence of having all three Triad components is up to 16%, two components is up to 27%, and having one of the components can be as high as 60% [11]. Low bone mineral density and menstrual challenges may not always be discernable early on, but changes in menstruation and impaired bone health should remain of utmost importance to clinicians. The ability to recognize the Triad should not only aim at treating the performance/energy needs, but rather the whole person. Examining energy demands and how someone is meeting them is merely a good start to unveiling what other areas need attention and care.

Exploring these components among individuals in a clinical setting can be difficult due to the variation in their presentation, and while we desire to develop individualized care, it can still be helpful to use a consistent method or validated approach. However, more than one assessment tool for the Triad exists [12], with debate on which is the more effective or appropriate tool. This may make it difficult for clinicians in the field to identify which is most suitable and how to adapt the tools to the resources they have. Thus, in this study, we will specifically investigate the use of the Triad Cumulative Risk Assessment (CRA) tool in performing artist. As clinicians grow in their understanding of the performing artist, there should be consideration for the focus of prevention and intervention of the Triad risk factors [8]. Therefore, the purpose of this study was to examine the use of the CRA tool and decision-making model for developing individualized medical plans for patients at risk for the Triad and to explore the dietary framework of collegiate dancers. These factors are often under-explored in the dance population, highlighting a gap within the literature and the importance of such research at this time. Specifically, we set out to investigate the following: (1) the Triad risk among collegiate dancers using the CRA tool, (2) return to play criteria, and (3) the macronutrient and micronutrient intake of collegiate dancers.

## 2. Materials and Methods

We utilized a retrospective study design within a university dance setting. This study was part of a larger study examining ED risk in collegiate female athletes and, therefore, follows a similar methodology for the data collected [13]; not all data are presented, and this study is a secondary analysis of the data. The secondary analysis also included going back to medical records to examine medical history and stress fracture history of participants. The study was conducted according to the guidelines of the Declaration of Helsinki and deemed exempt approval by the Institutional Review Board of University of South Carolina (cross-sectional study submission: Pro00019277; approval date: 28 September 2016; and for secondary analysis for retrospective study submission: Pro00099029; approval date: 29 January 2020).

### 2.1. Participants

Participants were female collegiate dancers (weight: 56.4 ± 7.0 kg; height: 165.3 ± 6.9 cm; age: 20.5 ± 3.6 years) recruited from the University of South Carolina Dance Company. Inclusion criteria included dancers to be actively participating with either ballet or contemporary/modern dances and between the ages of 18 and 35 years old. Dancers included students who majored or minored in dance from the Department of Theatre and Dance. Dancers in the company were required to take ballet and contemporary classes regardless of their style of focus, and all can perform in both ballet and contemporary shows. Those with a current injury keeping them from full participation (dance classes, rehearsals, performances) and those with known/diagnosed EDs were excluded from this study.

### 2.2. Cumulative Risk Assessment

The Cumulative Risk Assessment (CRA) is an assessment tool initiated by the Female Athlete Triad Coalition that examines the following components: LEA with or without ED, low body mass index (BMI), delayed menarche, oligomenorrhea and/or amenorrhea, low BMD, and stress reaction/fracture [14,15]. The assessment of each of those categories is what factors into the final risk score. Triad risk scores are defined as 0 = low risk, 1 = moderate risk, and 2 = high risk. For this study, the evaluation criteria or the assessments of each category were modified from the original [10] and are presented in Table 1. The construct validity of Table 1 was established by 6 healthcare professionals providing healthcare services to female athletes. Additionally, clinicians individually scored all dancers in each category. Upon individual scoring, three clinicians met to come to a consensus and clarify any scores that may have differed from the others. Once a consensus was reached, individual scores from each category were then summed to establish the participant’s cumulative Triad risk. Low cumulative scores are indicative of lower Triad risk and higher scores of higher risk. Final scores were associated with recommendations on return to play (RTP) or performance and were identified as either “fully cleared (0–1 point)”, “provisionally cleared (2–5 points)”, or “restricted from play (≥6 points)”. The CRA tool has different risk factor categories that clinicians are encouraged to address in determining the level of risk their patient falls. We have outlined how we achieved or conducted the assessment of that risk factor. The CRA tool has been used throughout the literature to help determine Triad risk and provide return to play (RTP) recommendations according to those risks [4,16]. The modified CRA tool created for our study can be found in Table 1.

#### 2.2.1. Previous Collected Data

Data from Torres-McGehee et al. [13] were used for secondary analysis. We obtained demographic data (e.g., age, race/ethnicity, etc.), dance history, and medical history (past health and injury and menstrual cycle). Anthropometric measurements included height, weight, body fat percentage, BMI, and fat-free mass. Eating disorder risk was calculated using the Eating Disorder Inventory-3 and Eating Disorder Inventory-3 Symptom Checklist. A 7-day dietary intake and exercise log/arm bands were used to determine energy availability, and bone mineral density was collected via dual energy X-ray absorptiometry (DEXA).

#### 2.2.2. LEA with or without an Eating Disorder

To assess dietary intake, each participant recorded a 7-day food log. Typically, a 2–3-day food log has been considered sufficient for assessment and replicated across the literature; therefore, a 7-day log provided us with sufficient information regarding the intake, eating habits, and general energy balance of our participants [17]. Food intake was also reported on the ESHA online food processor. ESHA is a nutritional analysis program used for research that allows the participant to input what they have eaten (i.e., the specific food and portion size). The reports established from the ESHA food processor were used to obtain all macronutrient and micronutrient profiles for each dancer, including all daily and cumulative intake of carbohydrates, proteins, fats, and a multitude of vitamins and minerals. All macronutrient and micronutrient intake for each participant was compared to “normal” daily recommended intake (DRI) for women 18–35 years old for each nutrient. The nutrient’s normal ranges were obtained through DRI values and literature exploring these same components [18]. Once all nutrient cutoff values were determined, they were sent to an expert in sports dietetics for review before implementation within this study. Each participant’s intake was compared to the normal recommended intake ranges to identify and categorize “low,” “within normal,” and “high” consumption.

Energy availability is a component that considers both exercise and dietary intake. More specifically, EA is calculated by taking energy intake (EI) minus exercise energy expenditure (EEE; EA = EI − EEE) and is expressed in kcal/kg/FFM (fat-free mass) [10,17]. Low energy availability is considered to be an EA below 30 kcals/kg/FFM [10]. To determine total daily energy expenditure (TDEE), EEE, and EA, each participant wore a fitness armband (SenseWear) every day for one week. Participants were instructed not to wear the armband in water or in performances. These measurements gave us a better understanding of the overall energy demands for each person and an outlook on the overall energy demands required from collegiate dance. Each participant self-reported all daily exercise into their physical activity log. This report included planned and intentional exercise/dance (e.g., things such as dance classes, dance rehearsals, outside exercise, performances, etc.). Participants provided detailed information regarding the extent of their exercise, duration, and what they were doing.

To better understand and assess the overall nutritional behaviors, perceptions, and pathogenic behaviors among our participants, participants completed the Eating Disorder Inventory-3 (EDI-3) [18]. The EDI-3 is a self-reported questionnaire to assist clinicians in revealing the presence of EDs or those at risk of EDs. The EDI-3 is comprised of 91 items with several composites ranging from ED risks to psychological components. The EDI-3 symptom checklist (EDI-3 SC) allows clinicians to evaluate how often certain symptoms (e.g., binge eating, laxative use, exercise to control weight, etc.) occur. The Diagnostic and Statistical Manual of Mental Disorders (DSM-5) defines and classifies health disorders to aid in the identification and treatment by clinicians. It provides an in-depth look at the many signs and symptoms that may be present with feeding and eating disorders. Eating disorders are classified as continuous disturbances in eating behaviors which result in altered amounts of consumption of food. By using the EDI-3 and EDI- SC self-reporting measures, we could understand how our dancers were engaging in the pathogenic behaviors associated with ED/DE [19].

#### 2.2.3. Body Mass Index, Low Bone Mineral Density, and Stress Fracture

Anthropometric measures were examined in various methods and used to capture BMI, which is defined as an individual’s weight divided by their height (kg/m^2^). Height was taken with a stadiometer height board (Shorr Productions, Olney, MD, USA) and weight with a Tanita scale (Tanita SC 331S, Tokyo, Japan). The Tanita scale considers the age, height, body type (athletic or standard), weight of clothing, and sex of the participant to provide detailed readings. Body composition (body fat percentage) was assessed using dual energy X-ray absorptiometry (DEXA) scan.

Bone mineral density (BMD) was also collected using dual X-ray absorptiometry (DEXA) scan. The DEXA scan uses X-rays to produce images providing details on the body’s bone mineral density (BMD) and mass [20]. A trained technician assessed all dancers using a full-body scan to gather complete data on the density of each dancer’s bones. Results are reported as a Z-score, which compares the results from each scan to that of women with similar age and body composition. We obtained each dancer’s history with stress fractures through access to the dancer’s medical records from their athletic trainer as well as their self-reported questionnaire highlighting any history with stress fractures.

#### 2.2.4. Menstrual History

Menstrual dysfunction or irregularities can range (i.e., delayed menarche, amenorrhea, and oligomenorrhea) and take time to manifest. We collected a variety of data on menstrual function regarding the dancer’s overall experience of missing cycles, the length of their absence, and if they happened during or out of season. All menstrual data were collected using a self-reported menstrual cycle questionnaire.

#### 2.2.5. Medical Record Data

Medical records were accessed using the electronic medical system (The Athletic Training System). All medical records were deidentified for data analysis and recoded with an identification number. In addition, anthropometric measurements, age, number of injuries along with location of injuries, previous/current dieting, menstrual history, and current medication were recorded.

### 2.3. Procedures and Protocols

Participants in this study were convenient samples recruited from the University of South Carolina Dance Company. Data collection took place during the fall (September–November) of 2016 and spring (January–February) of 2017, and secondary analysis data were collected in 2020. Initial data collected followed methodology and procedures outlined by Torres-McGehee et al. [13]. Data collection started with an informational session on the study aims and procedures. Those who were both willing to volunteer and met the criteria for the study were asked to sign a consent form prior to participation. At this time, the participants completed all physical measurements (e.g., height, weight, RMR, etc.) and completed questionnaires (demographics, the EDI-3, and EDI-SC). After the participant’s anthropometric measures were assessed, the participant was instructed on how to properly use the fitness armband, document dietary intake/activity, and enter diet in the ESHA food processor system online. Dancers then wore a fitness armband assessing energy expenditure over 7 days. Dancers were instructed to participate as normal in daily activities, dance classes, rehearsals, and performances. In addition to wearing the armband, each dancer was instructed to document all physical activity and food intake for the entire week. Medical records were used as supplemental information to classify each dancer using the Triad CRA.

### 2.4. Data Analysis

SPSS statistical software (Version 28; IBM Corp., IBM SPSS Statistics for Windows, Armonk, NY, USA) was used for all analyses. We used G*Power software (version 3.1.9.4; Franz Faul, Universität Kiel, Kiel, Germany) to calculate power. Using <0.05 and a large effect size (0.7), the power calculation indicated a sample size of 22 is needed for an estimated power of 0.9. Basic descriptive statistics were used for examining demographic information, the proportion of participants were identified for CRA risk and RTP, and each classification of the nutrients was assessed. CRA risk for RTP was classified as scores 0–1 (Full Clearance), 2.5 (Provisional/Limited Clearance), and ≥6 points (Restricted from Training/Competition Provisional or Disqualified). Energy availability was calculated as “EA = (EI − EEE)/FFM” and exercise energy expenditure EEE = duration (minutes) × ((METs × 3.5 × weight (kg))/200). LEA was defined as <30 kcal/kg^−1^ FFM for all dancers. To be considered at risk for an ED, participants must have at least 1 composite score rated as typical clinical or elevated clinical, meet the criteria or pathogenic behavior risk, or both.

## 3. Results

Of the 28 dancers who volunteered, all 28 participants were included in the CRA analysis, however, 2 did not complete the food log and expenditure (EI/EEE) portion of the study, thus all pertinent energy assessment results could not be captured for these 2 participants. Due to this, a final total of 26 dancers’ data were analyzed for energy assessment. Dance styles were broken into three categories: ballet (*n* = 20), contemporary (*n* = 7), and both ballet and contemporary (*n* = 1). The distribution of race among dancers were 96% self-identifying as white.

### 3.1. Cumulative Risk Assessment

Dancers averaged 3.5 ± 1.6 total score on the CRA tool. Based on these scores, the RTP outcomes were as follows: Full Clearance 7.1%, *n* = 2; Provisional/Limited Clearance 82.1%, *n* = 23; and Restricted/Medical Disqualification 10.7%, *n* = 3. These results would place those dancers at Provisional or Limited Clearance for RTP, meaning they should be monitored and assisted through treatment while they maintain some level of involvement in dance rehearsals. Those dancers with Restricted/Medical Disqualification inform the need to fully restrict dance involvement or medically disqualify a dancer and focus on recovery and health.

#### 3.1.1. LEA with or without an Eating Disorder

Overall energy assessment data can be found in Table 2. Risk for LEA was based on averages of the dancers’ EI and EEE over the week. In our sample, the mean EA of all 26 participants was 12.2 ± 11.3 kcals/kg FFM/day. When examining the EA breakdown and where the dancers fell along the spectrum (low = <30 kcal/kg/FFM, normal = within 30–45 kcal/kg/FFM, and above ≥45 kcal/kg/FFM), 69.2% (*n* = 18) were low and deemed at risk for LEA. Only 23.1% (*n* = 6) met their EA needs, and 7.7% (*n* = 2) exceeded them. Although on average 30.8% (*n* = 8) were not at risk, this does not mean that these dancers were not at risk at some point through the week. The mean days at risk for LEA was 5.1 ± 1.2 days, while the mean number of days exercised was 5.7 ± 1.0 days. Based on these findings, all 26 of the dancers were at risk of LEA roughly 50% of the week, with 15.4% (*n* = 4) at risk 50–75% of the week, and 84.6% (*n* = 22) at risk 75–100% of the week.

We used both the EDI-3 (Table 3) and the EDI-3 SC (Table 4) to determine the dancers’ ED risk. Overall, ED risk was 76.9% (*n* = 20) for ED/disordered eating behaviors. When looking at the tools separately, 69.2% (*n* = 18) were identified as at risk from the symptom checklist only and 38.5% (*n* = 10) were identified as at risk with the EDI-3 questionnaire only. Pathogenic behaviors (binging, purging, and dieting) were the highest reported behaviors (Table 3). Based on the EDI-SC risk, 42.3% (*n* = 11) of the dancers reported one pathogenic behavior and 38.4% (*n* = 10) reported two or more pathogenic behaviors. 

#### 3.1.2. Body Mass Index, Low Bone Mineral Density, and Stress Fracture

Anthropometric measurements and demographic results (height, weight, BMI, body fat%, etc.) are shown in Table 5. The difference on average between the dancer’s measured weight and what they identified as their ideal weight was 2.0 ± 1.5 kg. Overall, most dancers indicated an ideal weight that was lower than both the measured weight and what they self-reported as their actual weight.

While on average BMD was normal within our dancers, we did see three dancers with negative Z-scores who were also determined to be at risk for LEA. Table 6 represents BMD outcomes across the different segments. Within our sample, only four dancers reported having had a stress fracture in their time as a dancer.

### 3.2. Nutritional Behaviors

A complete breakdown of macronutrient and micronutrient/mineral values can be found in Table 7. It is worth noting that the dancers’ individual nutritional needs could exceed the basic requirements for women their age, but these data provide insight into whether each dancer minimally meets the standard need for the macronutrients and micronutrients assessed.

## 4. Discussion

### 4.1. Triad and Cumulative Risk Assessment

Our results revealed that all dancers had some form of Triad risk, primarily moderate risk, and recommendations for return to play were Provisional/Limited Clearance. This highlighted the importance of understanding the factors within the Triad and implementing assessments such as the CRA tool into actual practice. For our study specifically, health consequences seen within the Triad appear to be induced by low energy availability. Recognition of the Triad is just the beginning. As we know, patients can present with one or more of the components of the Triad and the cause can stem from a variety of reasons. Understanding the underlying cause of an individual’s Triad symptoms is crucial, as the presentation is not the same for every person. While the CRA tool is helpful for clinicians, it should be used strategically. There has been some debate on the use and implementation of the CRA to identify Triad risk due to room in the tool for personal interpretation [14,15]. This is why we modified our use of the original tool to encompass more ways to approach achieving the assessment of each category. The CRA tool has practical value, and its use provides clinical guidance surrounding decision making regarding patients’ Triad status and returning to performance. We found that our modifications of further defining aspects of the CRA tool allowed our research team to effectively score each dancer consistently. We were definitive in how each category was assessed in hopes that other clinicians, such as athletic trainers and physical therapists, can find applicability within their respective settings. Limitations still exist, because clinicians are subject to the resources they do or do not have available to them (i.e., DEXA scans).

Given that there are many variations in resources clinicians have access to, interprofessional relationships and collaborative efforts are vital in keeping the patient’s needs at the center of the identification and treatment. The CRA tool should be a starting approach to making informed clinical decisions that are adequately shaped to the needs of the patient. It should be based on factors such as the presence of certain signs and symptoms and the demands of the environment on the patient and aim to involve the patient in the goal-setting process or the development of recovery benchmarks. This collective view will allow the clinician to approach the patient’s condition with the right sensitivity and lead to desirable outcomes for the patient’s recovery. The CRA RTP guidelines are not hard set but rather used to help establish what may be the best next steps for the patient. Again, patient restrictions will be heavily influenced by how the condition is present for them individually, and the specific demands of the activities they specifically engage in. Considering that treatment plans will differ in patients for example those with LEA vs. LEA with ED, clinicians should be aware that these RTP guidelines are not hard-set rules but rather recommendations to influence direction.

#### Energy Outcomes and Eating Disorder Characteristics

Failing to consume the energy needed for the energy demands of both exercise and metabolic needs increases the risk for LEA [21]. Low EI ranges have been documented when caloric intake is less than 1800–2000 kcal/day for athletes [22]. The average EA for the dancers within our study was 12.2 ± 11.3 kcals/kg FFM/day, which is remarkably below the recommendation. Doyle-Lucas et al. compared dancers (ages 18–35 years old) to the female active control population and found that EI among dancers was significantly lower than the control group (1577 kcal/day vs. 2075 kcal/day) [23]. Additionally, among pre-professional dancers, Brown et al. discovered a higher average EI of 2428 kcal; however, EEE was 2784 kcal, resulting in EA falling below the cutoff of 26 kcal/kg FFM/day [24]. Findings from both studies, as with ours, suggest that low caloric intake impacts energy availability.

When comparing our dancers across other sports, varsity volleyball players had a mean EA of 42.5 kcal/kg FFM/day and an average EI of 3435 kcal/day [25]. Reed et al. [26] examined energy needs among 19 female collegiate NCAA Division I soccer players and found that LEA during the preseason, midseason, and postseason was observed in 26%, 33%, and 12% of the athletes, respectively. During midseason, the average EI was 1491 kcals/day for those with LEA and 2567 kcal/day for those without LEA. Overall, those with higher EA met the nutritional recommendations outlined by the ACSM [22]. Silva et al. [27] evaluated the dietary composition of elite rhythmic gymnasts before a competition and found that gymnasts had an average EI of 1629.2 ± 344.8 kcal/day and an average EEE of 695.9 kcal/day (ages 16–18 years old) and an average EI of 1802.9 ± 289.4 kcals/day and an average of EEE of 899.1 ± 222.4 kcals/day (ages 19–26 years old). LEA was present in 44.8% of the gymnasts [27]. From these reports we can see a few consistencies in that, generally, a lower EI was associated with LEA, and that across the board activities that were more aesthetic in nature such as dance and gymnastics tend to report lower EI relative to their needs identified by their EEE.

In our study, overall, ED risk was 76.9% (*n* = 20), with dieting as the most reported pathogenic behavior. This behavior showed to have major implications on the dancer’s daily food intake, which further impacted their nutritional framework. Eating disorders can be complex in nature with other psychological conditions present, thus there is not one cookie-cutter way to address its onset. Chronic levels of low caloric intake may also stem from unintentional reasons, such as lack of education on what is appropriate, time restraints that limit the preparation of meals and snacks for a long day of training and performances, or even financial reasons [28]. Therefore, it is important to know that it is not always that an athlete or performer is intentionally desiring major restrictions in their diet leading to disordered eating patterns and/or future eating disorders. However, it is an important factor to monitor, as small dietary changes can become obsessive or compulsive behaviors.

### 4.2. Nutritional Behaviors

As energy demands increase, nutritional changes must be considered as well as the difficulty in balancing the desired intake with training schedules and load [29]. As suspected, the dancers in our study consumed below the recommended carbohydrates, which seemed to be the primary reason for the overall low EI. Carbohydrate intake can improve the ability to sustain long-duration types of activities at moderate to high intensities, plays a role in delaying fatigue, and supports generating more adenosine triphosphate (ATP) [22]. It is suggested that due to the high energy required for the athletic population, consuming adequate carbohydrates should be the first aim of the “training diet” [22]. Like the results of carbohydrates, participants in our study significantly consumed below their needs for protein. Protein may have some benefits when combined with carbohydrates during long intermittent exercise periods but may be more beneficial post-exercise for repairing tissue [30]. Dancers in our study were considerably low in carb/protein intake as compared to other literature. Compared to the dancers in our study, pre-professional contemporary dancers, on average, met the recommendations for carbohydrates (5.0 g/kg/day), proteins (1.3 g/kg/day), and fats (34% of total energy intake) [24].

Our dancers were low in most key micronutrients examined. Vitamins and minerals help support metabolic processes and aid growth and energy conversion [7]. Micronutrient/mineral deficiencies can also lead to symptoms that have physical manifestations. Vitamin D, for example, plays a role in protein synthesis, inflammatory responses, immune support, and regulation of muscle function [31,32]. Symptoms of Vitamin D insufficiency include muscle weakness [31].

The International Association for Dance Medicine and Science (IADMS) has made suggestions on the amount of nutritional intake a dancer needs (55–60% carbs, 12–15% protein, and 20–30% fat) that are similar to that which is outlined in the ASCM (American College of Sports Medicine) guidelines for athletes [22]. However, it is important to note again that every dancer will vary in their overall nutritional needs from both a caloric and composition standpoint, and may require modifications as the intensity and frequency of performances change. It is common that when dietary strategies are implemented across active populations, it usually surrounds short-term performance enhancement. These considerations are great; however, clinicians must also examine using nutritional guidance for long-term health and wellness, specifically in women. A holistic view of the patient’s dietary framework allows clinicians to establish positive interactions and conversations around nutrition, which can improve patient-centered care. Healthcare providers, particularly athletic trainers who may be on site and working with patients daily, can consistently advocate for their patient’s well-being by establishing and cultivating a patient–provider relationship [33]. This is especially important in settings like the performing arts, where the culture can be one that negatively influences dancers to engage in poor eating behaviors.

Numerous dietary intervention strategies in sports have been utilized to support behavioral change, including education, enablement, training, environmental restructuring, modeling, and coercion [34]. Modes of implementing strategies also vary, such as delivering information through presentations, educational sessions, written information, handouts/worksheets, providing access to sport dietary products, and access to counseling services [34]. Nutritional needs and modifications are not one size fits all and can also vary within the same person depending on outside variables impacting their health at that time [30]. Typically, obtaining nutrients from a regular diet is sufficient; however, when there are known deficiencies, supplementation may be recommended. Individual variability is a key consideration when advising and educating patients on recommended intake and one reason why access to healthcare providers is so important, but often missing, in the dance population.

### 4.3. Limitations of the Study

Within our study, we could not control the frequency and duration that each dancer was in dance class, rehearsal, or partaking in other physical activities. However, dancers recruited from this study had very similar dance schedules to fulfill their degree requirements. For aesthetic purposes, none of the armbands could be worn during the dancers’ performances; therefore, no data were collected during that time, which could have impacted the overall energy expenditure outcomes we obtained. Finally, this is a retrospective study, so when using medical records, we did not have access to participants to follow up with medical records.

### 4.4. Clinical Significance and Future Research

Our study identified overall risk for the Triad, engaging in pathogenic behaviors, and nutritional deficiencies in the dance population. If left untreated, the physiological manifestations of these behaviors affect the dancers’ performance and overall wellness. There is a high need to implement healthcare services (i.e., athletic training, physical therapy, physicians, dieticians, etc.) in the dance population for prevention and treatment of the components of the Triad along with the nutritional deficiencies that may also manifest.

Future investigations should continue to examine the factors leading to these disorders, and examine how energy deficits, due to nutritional imbalances, impact recovery and metabolic changes in performing artists. Within the variables of the Triad and nutrition, future research should aim to make comparisons between the various styles of dance, training, and performance, as well as explore these factors among male artists to develop more literature surrounding the male Triad. This can be influential in discovering any differences that may exist between training and performance nutrition needs across gender. In settings such as dance there may be several compounding factors, such as the environment and culture, influence from instructors, and mental health concerns, which should also be explored in upcoming literature.

## 5. Conclusions

Our study identified that a majority of dancers were at moderate Triad risk, primarily with LEA and some with ED risk, with recommendations for RTP with Provisional/Limited Clearance. Energy deficiencies were prevalent among these collegiate dancers, and their nutritional intake was substantially deficient in both macro- and micronutrient components. Although LEA is only one component of the Triad, it is understood that each aspect of the Triad is no longer needed to occur in conjunction with one another for a dancer to be affected by the physiological consequences. Here, we saw how LEA greatly influenced the presentation of the Triad in our dancers, but it will be vital for clinicians to have individualized evaluation plans for their patients. The CRA tool provides a great framework for the assessment process of the Triad that should be used to further adapt a more tailored plan around the patient’s greatest need. The importance of our findings is critical. The dietary framework of performing artists, much like athletes in traditional sports, is vital in maintaining enough fuel to sustain energy and maintain long-term health. We should continue exploring the Triad’s susceptibility within the performing arts. This will bring about more awareness of the demands within dance but allow clinicians to expand their ability to recognize, treat, and prevent the major health concerns that are associated with Triad deficiencies among this population. Tools such as the Triad CRA tool and their RTP resources should be considered for clinical guidance. However, clinicians should be mindful that constructing individual timelines for recovery while also involving the patient in the development of milestones is equally important. When possible, we should attempt to introduce a multidisciplinary medical team from a variety of professions (i.e., physicians, athletic trainers, dieticians, and sports psychologists/counselors) who will collaborate in delivering successful well-rounded treatment and maintain an inclusive and patient-centered approach for management of a patient with Triad and/or nutritional deficiencies.

## Figures and Tables

**Table 1:** Application of Triad CRA for screening.

Risk Factors	Study Data	Risk Scoring *
LEA with or without an ED/DE	EDI-3 EDI-3 SC LEA	Low → no dietary restrictions; no LEA (EA ≥ 45 kcal/kg/FFM) and no ED risk Moderate → EA (30–45 kcal/kg/FFM) and/or ED risk High → LEA (≤30 kcal/kg/FFM) with and/or without an ED risk
Low BMI	Measured height and weight to calculate BMI	Low → BMI ≥ 18.5 OR ≥ 90% estimated weight or weight stable Moderate → BMI 17.5 < 18.5 OR < 90% estimated weight High BMI ≤ 17.5 OR < 85% estimated weight
Delayed Menarche	Menstrual cycle survey	Low → menarche < 15 years old Moderate → menarche 15 to <16 years old High → menarche ≥ 16 years old
Amenorrhea/Oligomenorrhea	Menstrual cycle survey Medical records	Low → 9 menses in 12 months Moderate → 6–9 menses in 12 months High → <6 menses in 12 months
Low BMD	DEXA	Low → Z-score ≥ −1.0 Moderate → Z-score −1.0 < −2.0 Non-weight-bearing sports: crew, cycling, horseback riding, sailing, scuba diving, swimming, and water polo Participates in any form of weight-bearing activity as supplemental training for a non-weight-bearing sport (e.g., a swimmer performing resistance training) High → Z-score ≤ −2.0
Stress Reaction/Fracture	Medical records	Low → None Moderate → self-report or through medical records 1 fracture or previous stress fracture history High → ≥1 high risk of trabecular bone sites (lumbar spine, femoral neck, sacrum, and pelvis)

Abbreviations: BMI = body mass index, DE = disordered eating, DEXA = dual energy X-ray absorptiometry, ED = eating disorder, EDI-3 = Eating Disorder Inventory-3, EDI-3 SC = Eating Disorder Inventory-3 Symptom Checklist. * Scoring for low risk = 0 points, moderate risk = 1 point, and high risk = 2 points.

**Table 2:** Energy need assessments (*n* = 26).

Energy Needs Assessment	M ± SD
Resting metabolic rate (kcals)	1155.8 ± 206.5
Energy intake (kcals)	1473.9 ± 312.5
Exercise energy expenditure (kcals)	810.9 ± 408.1
Energy availability (kcals/kg^−1^ FFM)	12.2 ± 11.3
Total daily energy expenditure (kcals)	2297.1 ± 127.3

Values are presented as mean ± Standard Deviation.

**Table 3:** Eating disorder scores (from EDI-3) among dancers (*n* = 28).

	Raw Scores M ± SD	Low Clinical % (*n*)	Typical Clinical % (*n*)	Elevated Clinical % (*n*)
ED Risk Scale				
Drive for Thinness	8.50 ± 7.21	84.6% (22)	15.4% (4)	0% (0)
Bulimia	2.84 ± 3.41	80.8% (21)	19.2% (5)	0% (0)
Body Dissatisfaction	14.26 ± 9.14	80.8% (21)	15.4% (4)	3.8% (1)
ED Risk Composite	107.115± 21.25	92.3% (24)	7.7% (2)	0% (0)
Psychological Scale				
Low Self-Esteem	3.76 ± 3.58	88.5% (23)	11.5% (3)	0% (0)
Personal Alienation	4.30 ± 3.88	88.5% (23)	11.5% (3)	0% (0)
Interpersonal Insecurity	6.11± 4.38	65.4% (17)	30.8% (8)	3.8% (1)
Interpersonal Alienation	4.19 ± 4.089	76.9% (20)	15.4% (4)	7.7% (2)
Interceptive Deficits	4.19 ± 4.43	92.3% (22)	7.7% (2)	0% (0)
Emotional Dysregulation	1.65 ± 2.208	84.6% (22)	15.4% (4)	0% (0)
Perfectionism	12.00 ± 4.54	34.6% (9)	42.3% (11)	23.1% (6)
Asceticism	5.46 ± 3.44	80.8% (21)	19.2% (5)	0% (0)
Maturity Fears	6.26 ± 3.41	42.3% (11)	53.8% (14)	3.8% (1)
Composite				
Ineffectiveness Composite	69.76 ± 11.56	92.3% (24)	7.7% (2)	0% (0)
Interpersonal Problems Composite	79.76 ± 13.21	76.9% (20)	19.2% (5)	3.8% (1)
Affective Problems Composite	72.84 ± 8.11	96.2% (25)	3.8% (1)	0% (0)
Over control Composite	84.00 ± 11.56	69.2% (18)	26.9% (7)	3.8% (1)
General Psychological Maladjustment	349.73 ± 37.74	88.5% (23)	11.5% (3)	0% (0)

Abbreviations: ED = eating disorder. Raw scores presented as mean ± Standard Deviation and risk as % (*n*).

**Table 4:** Eating disorder pathogenic behaviors (from EDI-3 SC) among dancers (*n* = 28).

	All Data (*n*)	All Data (%)
Eating Behaviors		
Dieting	18	69.2%
Binge eating	5	19.2%
Purging	7	26.9%
Laxatives	0	0%
Diet pills	2	7.7%
Diuretics	1	3.8%
Exercise to Control Weight		
0% of time	0	0%
<25% of time	4	15.4%
25–50% of time	15	57.7%
More than 75% of time	7	26.9%
100% of time	0	0%

Abbreviations: EDI-3 SC = Eating Disorder Inventory-3 Symptom Checklist. Data are presented in frequency (*n*) and percent (%).

**Table 5:** Self-reported and measured physical measurements for dancers (*n* = 28).

Body Composition	M ± SD
Measured height (cm)	165.3 ± 6.9
Measured weight (kg)	56.4 ± 7.0
Ideal weight (kg)	54.4 ± 5.5
BMI (kg/m^2^)	20.6 ± 1.8
DEXA body fat (%)	28.0 ± 3.7
Fat-free mass (kg)	45.6 ± 4.4

Abbreviations: BMI = body mass index, DEXA = dual energy X-ray absorptiometry. Values are presented as mean ± Standard Deviation.

**Table 6:** Bone mineral density scores for dancers (*n* = 28).

Bone Mineral Density	M ± SD
Total Z-score	1.1 ± 0.7
Total score (g/cm^2^)	1.1 ± 0.0
Legs (g/cm^2^)	1.1 ± 0.0
Spine (g/cm^2^)	1.0 ± 0.1
Pelvis (g/cm^2^)	1.1 ± 0.1

Values are presented as mean ± Standard Deviation.

**Table 7:** Nutritional intake for 1 week among dancers (*n* = 26).

	Low % (*n*)	Within Normal % (*n*)	High % (*n*)
Macronutrients			
Proteins (g)	92.3% (24)	7.7% (2)	0
Carbohydrates (g)	96.2% (25)	3.8% (1)	0
Fat	19.2% (5)	61.5% (16)	19.2% (5)
Saturated Fat	0	80.8% (21)	19.2% (5)
Micronutrients			
Calcium (mg)	96.2% (25)	3.8% (1)	0
Iron (mg)	100% (26)	0	0
Vitamin D (IU)	100% (26)	0	0
Vitamin C (mg)	61.5% (16)	7.7% (2)	30.8% (8)
Vitamin B 6 (mg)	100% (26)	0	0
Vitamin B 12 (mcg)	100% (26)	0	0
Vitamin E (mg)	100% (26)	0	0
Vitamin K (mcg)	61.5% (16)	7.7% (2)	30.8% (8)
Folate (mcg)	11% (3)	0	88.5% (23)
Magnesium (mg)	0	0	100% (26)
Phosphorus (mg)	0	0	100% (26)
Zinc (mg)	100% (26)	0	0
Sodium (mg)	0	0	100% (26)
Potassium (mg)	0	0	100% (26)
Total Fiber (g)	84.6% (22)	0	15.4 (4)

## Data Availability

The data presented in this study are available on request from the corresponding author. The data are not publicly available due to IRB protections.
